# Mitral and aortic valve regurgitation following surgical and transcatheter perimembranous ventricular septal defect closure in children and adolescents: midterm outcomes

**DOI:** 10.1186/s12872-022-02757-9

**Published:** 2022-07-15

**Authors:** Mohammadreza Edraki, Mohammadjavad Nobahkti, Amir Naghshzan, Hamid Amoozgar, Ahmadali Amirghofran, Bahram Ghasemzadeh, Elahe Nirooie, Nima Mehdizadegan, Hamid Mohammadi, Kambiz Keshavarz

**Affiliations:** 1 Cardiovascular and Neonatology Research Center, School of Medicine, Shiraz University of Medical Sciences, Shiraz, Iran; 2 Cardiac Surgery Department, School of Medicine, Shiraz University of Medical Sciences, Shiraz, Iran; 3 Social Determinants of Health Research Center, Yasuj University of Medical Sciences, Yasuj, Iran; 4 Namazi Hospital, Shiraz, Iran

**Keywords:** Ventricular septal defect, Surgical closure, Percutaneous closure, Amplatzer, Mitral regurgitation, Aortic regurgitation

## Abstract

**Background:**

Closure of perimembranous ventricular septal defects (pmVSD), either surgical or percutaneous, might improve or cause new-onset mitral regurgitation (MR) and aortic regurgitation (AR). We aimed to evaluate the changes in MR and AR after pmVSD closure by these two methods.

**Material and method:**

We performed a comparative retrospective data review of all pediatric patients with pmVSDs treated at our institution with surgical or antegrade percutaneous methods from 2014 to 2019 and 146 consecutive patients under 18 years were enrolled. We closely looked at the mitral and aortic valve function after repair. Included patients had no or lower than moderate aortic valve prolapse and baseline normal mitral or aortic valve function or less than moderate MR or AR.

**Results:**

Out of 146 patients, 83 (57%) pmVSDs were closed percutaneously**,** and 63 (43%) pmVSDs were closed surgically. 80 and 62 patients were included for MR evaluation, and 81 and 62 patients for AR evaluation in percutaneous and surgical groups. The mean follow-up time was 40.48 ± 21.59 months in the surgery group and 20.44 ± 18.66 months in the transcatheter group. Mild to moderate degrees of MR and AR did not change or decreased in most patients. In detail, MR of 70% and AR of 50% of the valves were resolved or decreased in both groups. 13% of patients with no MR developed trivial to mild MR, and 10% of patients with no AR showed mild to moderate AR after pmVSD closure in both methods. There was no significant difference between the two methods regarding emerging new regurgitation or change in the severity of the previous regurgitation.

**Conclusion:**

pmVSD closure usually improves mild to moderate MR and AR to a nearly similar extent in both percutaneous and surgical methods among children and adolescents. It might lead to the onset of new MR or AR in cases with no regurgitation.

**Supplementary Information:**

The online version contains supplementary material available at 10.1186/s12872-022-02757-9.

## Introduction:

In recent years, transcatheter pmVSD closure with various techniques has been proposed due to the non-invasive nature of the procedure [1, 2]. The transcatheter method is usually used for small to medium-sized pmVSD closure, while surgical closure usually is considered for larger-sized pmVSDs. The transcatheter method is recommended if the subaortic rim between the pmVSD and the aortic valve annulus is more than 2 mm [1, 2]. Choosing the percutaneous method for patients with pmVSDs accompanied by MR or AR is debatable due to inadequate information.some patients without MR and AR who undergo surgical or percutaneous pmVSD closure may present with MR or AR during the follow-up. This study aimed to evaluate and compare the fate of aortic and mitral valve function and competence after pmVSD closure whether using surgical or antegrade transcatheter techniques.

## Material and methods

In this retrospective study, 146 consecutive patients under 18 years were enrolled from July 2014 to September 2019 in two teaching hospitals of Shiraz University of Medical Sciences, Shiraz, Iran. Included patients had no MR and AR, or lower than moderate baseline MR and AR before closure, no aortic valve prolapse, or lower than moderate prolapse of the non-coronary or right coronary cusps into the pmVSDs before treatment. The aortic rim of most pmVSDs was more than 2 mm and was less than 2 mm in a few. Inclusion criteria for pmVSD closure were defined as left to right shunt from the pmVSDs in the presence of one following criteria: QP / QS ≥ 2, cardiothoracic ratio ≥ 0.55 on standard chest X-ray, left atrium to aortic diameter ≥ 1.5 in the long axis echocardiography view, left ventricular dilatation defined as left ventricular end-diastolic volume ≥ 2 z-score, pulmonary artery hypertension, more than six lower respiratory tract infection in the preceding year, progressive MR or AR, or failure to thrive[2]. For patients with pulmonary artery hypertension, pmVSD closure was done if pulmonary vascular resistance was less than 6 Wood Unit. M^2^ [3].The patients who developed atrioventricular block, residual VSD shunt after closure, and mitral or aortic valve disorders not related to pmVSDs were excluded from our study. The patients were followed by two-dimensional transthoracic echocardiography before the closure, early after the closure, and during the serial follow-ups every six months. The echocardiographic data were analyzed in two sections 1–7 days after the closure and more than six months after the closure.

### pmVSD closure protocol

The antegrade percutaneous closure was chosen according to the site and size of the pmVSDs. If the pmVSD was small to medium-sized and MR or AR were none or mild to moderate, the transcatheter method was preferred, and the surgical closure was reserved for medium to large-sized pmVSDs or cases with more than mild to moderate MR or AR [4]. The VSD sizing was adjusted to the patient's weight and size of the aortic annulus. The size of VSD in echocardiography was considered small if it was less than one-third of the size of the aortic valve annulus, medium if it was one-third to half of the annulus, and large if it was more than half of the annulus [5, 6]. The percutaneous antegrade technique of the pmVSD closure is shown in Additional file 1: Video S1.

The entry of the occluders toward the pmVSDs was from the femoral veins or the superior vena cava, and the size of the occluders was usually chosen 2 mm larger than the maximum defects diameters in diastole.

In the surgical method, if the patients had more than mild MR, ring annuloplasty was done for mitral valve repair, and if they had more than mild AR, central plication was the method of choice to repair the aortic valve [7, 8].

The ethics committee of Shiraz University of Medical Sciences approved the study based on the Helsinki Agreement with the code number IR.sums.med.rec.1398.43, and written consent was obtained from parents or guardians before the procedures.

### The occluders

**Fig. 1:**
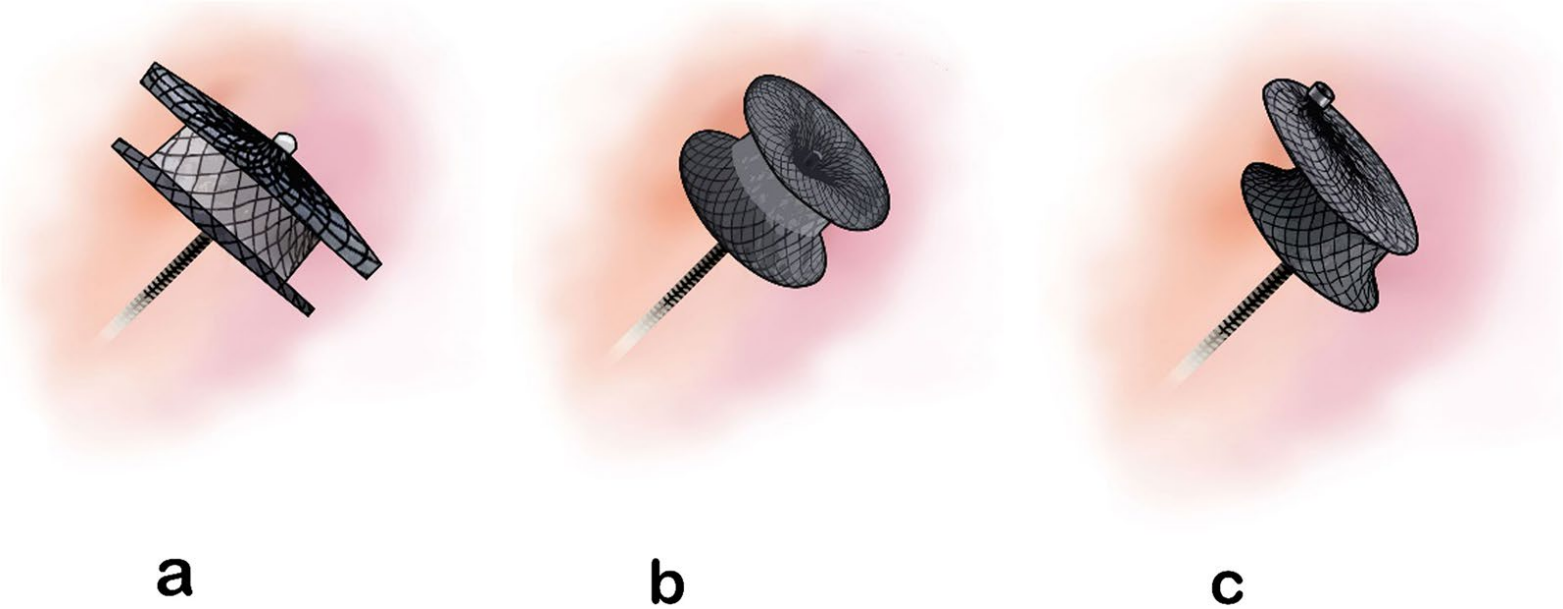
Different types of VSD Amplatzers.** a** symmetric perimembranous type.** b** muscular type.** c** perimembranous zero-edge type

Transcatheter pmVSD closure was done for VSDs with more than 2 mm aortic rim using symmetric pmVSD Amplatzer (Fig. 1a), asymmetric pmVSD Amplatzer, muscular VSD Amplatzer (Fig. 1b), Amplatzer duct occluder type I, and short shank Occlutech brand Amplatzer duct occluders. Eccentric zero-edge VSD occluders (Fig. 1c) were also used to close pmVSDs with less than 2 mm aortic rims.

### Echocardiography

The standard guidelines of transthoracic echocardiography were used [9, 10]. The parameters of M-mode, two-dimensional, and color Doppler methods were recorded with a Samsung HS70 echo machine [11–15] and stored in the database system of our institute. The location and size of pmVSDs were assessed in different standard echocardiographic views. MR classification included four categories: no, trivial, mild, and mild to moderate, and the AR classification included three categories: no, trivial to mild, and mild to moderate. We defined these two classifications to categorize valve regurgitations more easily. The best views for MR assessment were four-chamber and long axis. The MR degree was determined on the volume of color Doppler blood jet: if it was up to one-third of the left atrium, it was considered mild, up to 2/3 of the left atrium moderate, and the higher was considered severe [16, 17]. Aortic valve annulus and vena contracta dimension were recorded in long-axis view. Then vena contracta/aortic annulus ratio index was calculated, and < 5%, 5–10%, and > 10–20% were considered trivial to mild, mild to moderate, and moderate AR, respectively [18].

All patients in surgery and percutaneous groups were stratified into two groups based on the presence or absence of MR or AR before the pmVSD closure. Then, the new onset MR or AR and the changes of previous regurgitations after the closure were followed with echocardiography.

### Statistical analysis

In this paper, Continuous quantitative variables are shown by mean ± standard deviation and nominal variables by frequency and percentage. Chi-square test was used to compare two groups of surgery and transcatheter in terms of gender, age, weight, and pmVSD size, as well as echocardiography data like MR and AR. The Friedman statistical test was used to compare MR and AR changes before and during the follow-up. A p-value less than 0.05 was considered significant. Analyzes were performed by IBM SPSS statistics 25 software.

## Results

**Fig. 2:**
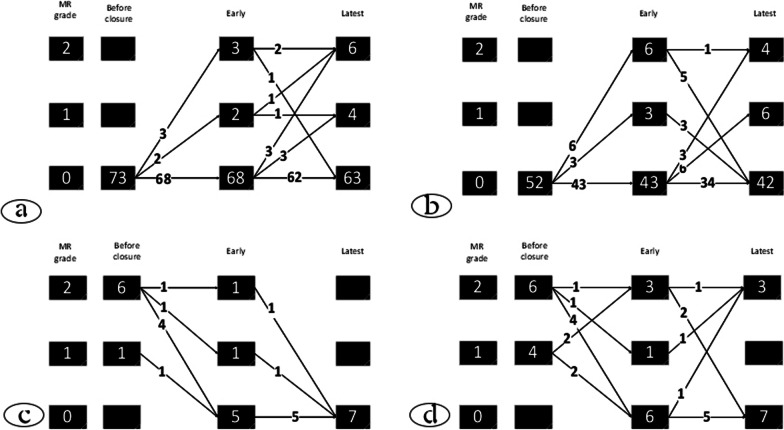
Evolution of MR in all patients: **a** The degree of MR after percutaneous pmVSD closure in the patients without MR before the closure. **b** The degree of MR after surgical pmVSD repair in the patients without MR before the operation. **c** The degree of MR after percutaneous pmVSD closure in the patients with MR before the closure. **d** The degree of MR after surgical VSD repair in the patients with MR before the operation. Grade 0 = No MR; Grade 1 = trivial MR; Grade 2 = mild MR; MR = mitral regurgitation

**Fig. 3:**
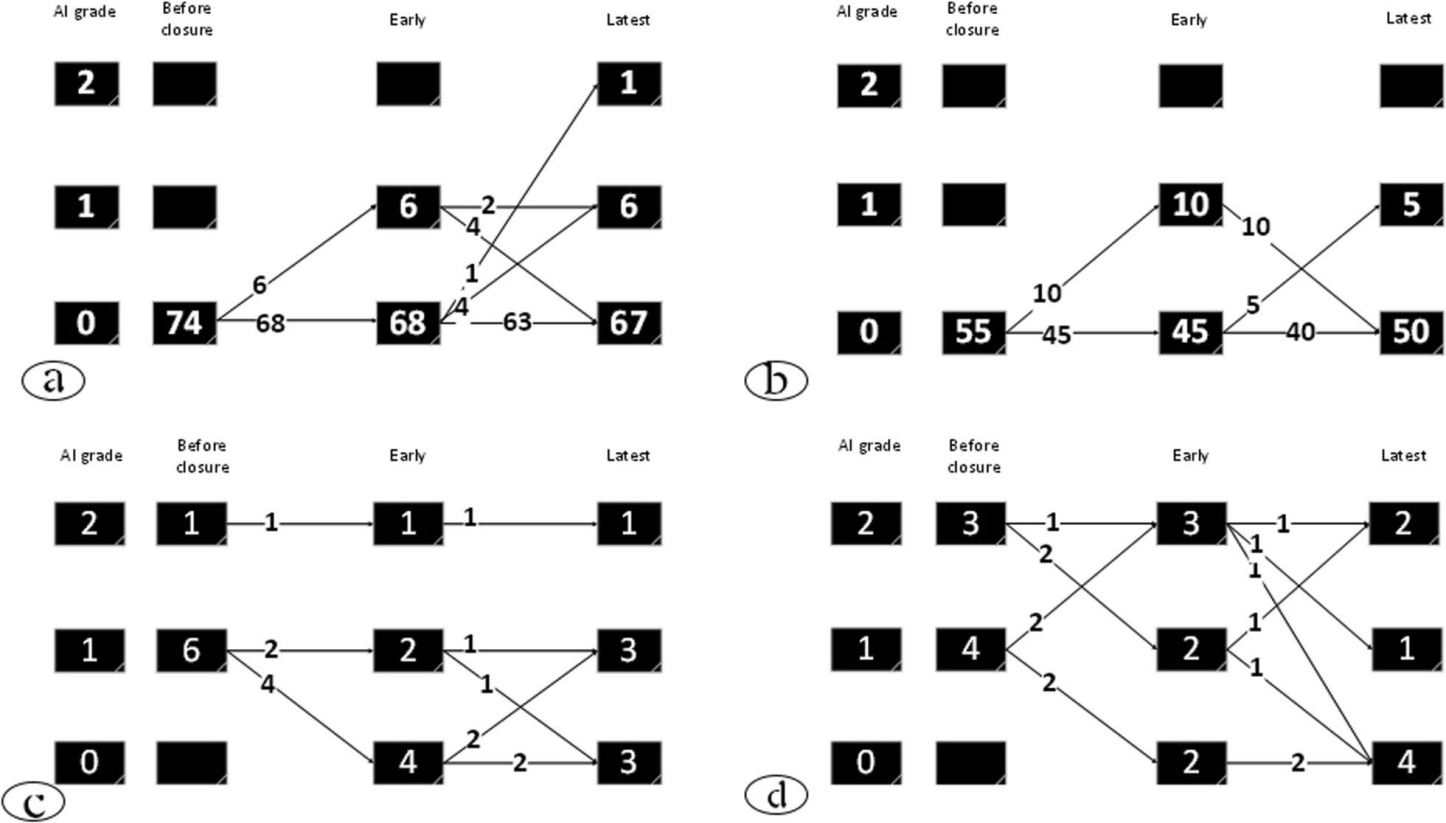
Evolution of AR in all patients: **a** The degree of AR after percutaneous pmVSD closure in the patients without AR before the closure. **b** The degree of AR after surgical pmVSD repair in the patients without AR before the operation. **c** The degree of AR after percutaneous VSD closure in the patients with AR before the closure. **d** The degree of AR after surgical VSD repair in the patients with AR before the operation. Grade 0 = No AR; Grade 1 = trivial to mild AR; Grade 2 = mild to moderate AR; AR = aortic regurgitation

In this study, our population consisted of 146 children and adolescents who underwent VSD closure. The mean follow-up time was 40.48 ± 21.59 months in the surgery group and 20.44 ± 18.66 months in the transcatheter group. 63 (43%) VSDs were closed surgically, and 83 (57%) pmVSDs were closed percutaneously. Table 1 shows the demographic characteristics of both groups. There was no gender difference between the two groups, but the patients' age and weight in the surgical group were lower than in the transcatheter group. The degree and incidence of MR and AR before the closure were statistically the same between these two groups (Table 1). According to Figs. 2 and 3, the changes in pre-procedure MR or AR after the closure were almost the same in both groups.

**Table 1:** Demographic data and Basic echocardiographic findings

Characteristics	Transcatheter	Surgery	p-value
Patients number	83	63	
Female/ male (%)	44 (53%)/39 (47%)	39 (61.9%)/24 (38.1%)	0.312
Age (years)- mean ± SD, range	5.77 ± 5.37 (0.4–18)	1.89 ± 2.71 (0.16–19)	< 0.001
Weight (kg)- mean ± SD, range	18.13 ± 13.83 (5–60)	8.36 ± 5.46 (3.5–38)	< 0.001
VSD size (mm)- mean ± SD, range	4.90 ± 3.45 (1–11)	7.39 ± 5.32 (2.3–17)	< 0.001
pmVSD (number)	83	63	0.043
Mitral regurgitation			
No	73 (88%)	52 (83.9%)	0.231
Trivial	1 (1.2%)	4 (6.5%)	
Mild	6 (7.2%)	5 (8.1%)	
Moderate	0	1 (2%)	
Aortic regurgitation			
No	74 (89.2%)	55 (88.7%)	0.494
Trivial-mild	6 (7.2%)	4 (6.5%)	
Mild-moderate	1 (1.2%)	3 (4.8%)	
EF% (mean ± SD)	73.25 ± 3.60	72.17 ± 6.79	0.248

SD: standard deviation, pmVSD: perimembranous ventricular septal defect, EF: Ejection fraction

### MR changes in percutaneous and surgery groups

Out of 83 transcatheter and 63 surgically treated patients, 80 and 62 patients were enrolled, respectively. Figure 2 shows the onset and the trend of MR degree in either group in patients with or without MR before closure.

MR was lower than moderate in both groups, and only one patient had more than mild (moderate) MR before closure whose pmVSD was closed with surgical method, and MR downgraded. Figure 2 does not mention this case to make the figure more comprehensible.

As shown in Fig. 2a in the transcatheter group, 73 (88%) patients had no MR before pmVSD closure. However, at the follow-up of these 73 cases, five patients showed trivial to mild MR during the early phase, five other patients showed trivial to mild MR after the early stage, and a total of 13% developed MR, which was at most mild. Figure 2c shows seven patients (10%) in the transcatheter group who had pretreatment MR, which was eliminated at the late follow-up.

In the surgery group, as shown in Fig. 2b, 52 patients had no MR before the operation, of whom 9 cases progressed to trivial to mild MR in the early phase, of which eight of them resolved in the later follow-up. However, nine other cases showed trivial to mild MR at the late follow-up, and a total of 15% developed new-onset trivial to mild MR. In the surgery group, As shown in Fig. 2d, 10 (19%) patients had previous MR, which was eliminated in 7 at the follow-up, increased in 2, and did not change in 1.

None of the patients without pretreatment MR developed regurgitation more than a mild degree in either group. In summary, about 13% of patients who did not have MR before the treatment developed trivial to mild MR following the closure in both methods. Of those with pretreatment MRs, about 70% of the MRs resolved or decreased during the follow-ups, and the remaining 30% did not change.

### AR changes in intervention and surgery groups

Of 83 transcatheter and 63 surgery patients, 81 and 62 had inclusion criteria for evaluating AR changes, respectively. Figure 3 shows the onset or trend of AR grade in both groups. As shown in Fig. 3a in the transcatheter group, 74 patients had no AR before the pmVSD closure, of whom 6 (8%) cases developed trivial to mild AR in the early phase, and one patient developed mild to moderate AR at the late follow-up. Thus, 7 (10%) patients without AR treated by the transcatheter method showed some degrees of AR at the follow-up. Figure 3b shows that out of 55 surgically treated patients without previous AR, 10 (18%) developed AR early after the operation, which all disappeared at the late follow-up. 5 (9%) cases did not have any AR in the early phase, but they showed mild to moderate AR in the late phase. 5 (9%) of all developed mild to moderate AR at the follow-up. Figure 3c shows that 7 (9%) patients had previous AR in the transcatheter group, eliminating in 3 patients and remaining in 4 almost the same as before. As shown in Fig. 3d, seven (7%) patients had AR before the surgery, and it was eliminated in 4 patients at the follow-up and remained in 3. None of the patients without AR before closure developed more than mild to moderate degrees of AR.

In summary, about 10% of the patients who did not have AR before pmVSD closure developed trivial to mild AR in both methods. About 50% of pretreatment ARs were resolved or decreased at the follow-up in either group. The remaining 50% did not change at all.

**Fig. 4:**
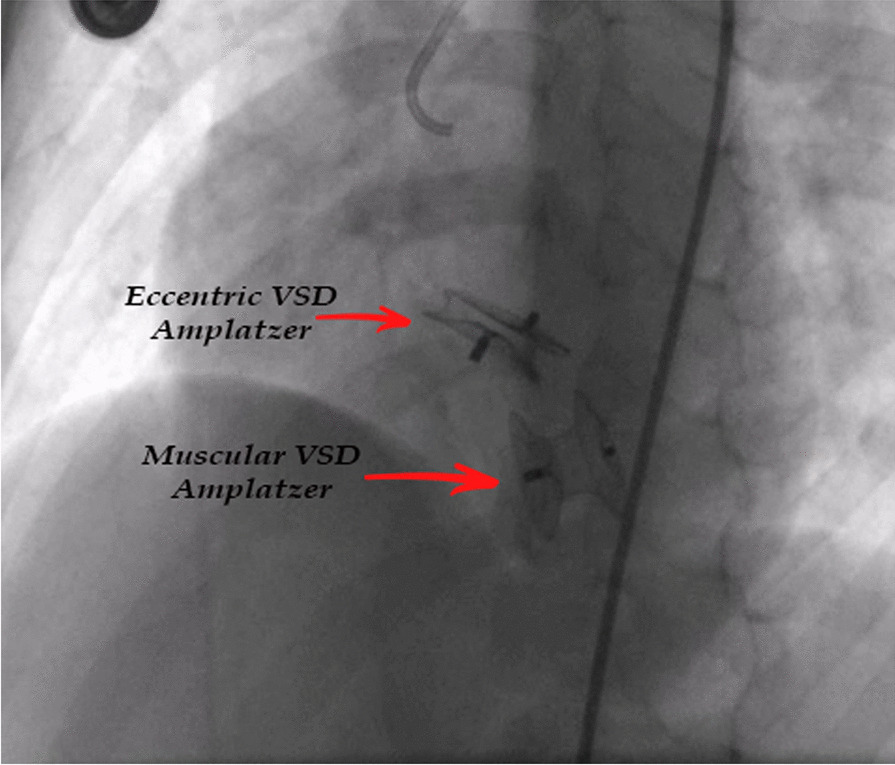
One of our patients who underwent a pmVSD closure with eccentric zero-edge VSD Amplatzer and a muscular VSD closure with a muscular VSD Amplatzer

In our study, pmVSD closure was done for four patients with mild aortic valve prolapse and less than 2 mm aortic rim with eccentric zero-edge pmVSD Amplatzer (Fig. 4). 1 of 4 patients did not have AR before and after the closure, and the other 3 had trivial to mild AR before and after the closure. The grade of AR changed during the follow-up in none of them.

### Friedman test

The Friedman test was used to compare degrees of MR and AR before, and early and midterm following VSD closure, and Table 2 shows that MR and AR changes were significantly associated with transcatheter or surgery methods.

**Table 2:** Friedman test result to compare changes in mitral and aortic regurgitation after percutaneous and surgical methods for VSD closure

Characteristics	Percutaneous method		Surgical method	
	Statistic	*p* value	Statistic	*p* value
Mitral regurgitation changes before and after VSD closure				
Existence of MR before procedures	12.09	0.002	8.60	0.014
No MR before procedures	10.45	0.005	10.11	0.006
Aortic regurgitation changes before and after VSD closure				
Existence of AR before procedures	5.20	0.074	3.60	0.165
No AR before procedures	7.81	0.02	10.00	0.07

AR = aortic regurgitation; MR = mitral regurgitation; VSD = ventricular septal defect

## Discussion

Given that VSD is a common heart anomaly and many cases require closure, it is essential to study the side effects of different closure methods and their impact on the heart valves. The factors influencing the choice of the surgical or transcatheter method vary. Valvar pathology, especially mitral and aortic valves, plays a pivotal role. In this study, our goal was to assess the development or changes of MR and AR in children and adolescents after surgical and transcatheter pmVSD closure.

The patients' age and weight in the surgical group of our study were lower than in the transcatheter group. This difference between the age and the weight of the two groups is related to the indications of VSD closure and our center's strategy for opting for the suitable VSD closure method.

Several studies were performed to determine changes in MR degree after pmVSD closure. Some of them showed the effectiveness of VSD closure on reducing the MR degree in the surgical method. They declared if MR is due to mitral valve annulus dilatation, the mitral valve repair is not required at the time of VSD closure [19–22]. While other studies recommend the mitral valve repair with any degree of MR [19]. Our study showed improvement of mild to moderate MRs after VSD closure, even though mitral valve repair was not performed.

In one study, the severity of MR decreased after the pmVSD closure by the transcatheter method in children [3]. The author believes that with the closure of VSD, hemodynamic improvement, removal of the left to right shunt, and reduction of the left ventricular size occur, and thus MR decreases.

In another study, the treatment strategy for MR was different. In addition to the degree of MR, other quantitative criteria such as the z-score of the mitral valve ring and the mean diastolic left ventricular diameter were considered. They suggested mitral valve repair at the time of surgery when MR is more than moderate in the presence of abnormal mentioned quantitative factors [19].

Hwa et al. studied the degree of MR after surgical VSD closure without mitral valve repair in children, and based on the mentioned annular dilatation as the mechanism of MR; they stated the improvement of MR could occur after the reduced left ventricular dilatation [21]. In this study, mild to moderate MR decreased in 50% of the patients in the first month after the pmVSD closure, and resolved within a year, and in 50% of the patients and the presence of moderate to severe MR the improvement was delayed, and most of them were entirely resolved or decreased to less than moderate after a year [21]. According to this study, there was no need to do a systematic repair of mitral valve surgery even with moderate MR. In another study on 60 patients with VSD and MR underwent surgical treatment, Finally authors showed simultaneous MVR has no benefits simultaneous MVR provided no advantage over that of isolated VSD closure [23].

In another article, the researcher investigated the MR degree in infants with VSD and found that MR may improve after surgical closure without mitral valve repair. They recommended mitral valve repair only for more than moderate MR [23]. In our study, mild to moderate MR was reduced in patients without mitral valve repair following either method.

It has been raised that VSD closure with surgical or interventional methods might lead to MR, as the complication of the therapies. Regarding some studies, two theories have been proposed to justify the development, sustainability, or increase of the MR degree after the treatment. One is attributed to the complications of aortic cross-clamping during the surgery related to the stunning cardiac phenomenon that occurs early after the operation [24, 25]. The second theory explains mechanical or hypoxic damage to the papillary muscles or mitral valve apparatus during VSD closure [26]. In the transcatheter closure, the primary mechanism of MR increase is the interference of the Amplatzer disk with chordae tendineae [27].

In our study, according to the incidence of a significant percentage of MR during the early post-operation phase and its rapid recovery process, the stunning heart phenomenon could be regarded as the cause. Based on the findings of our study and previous studies, it is crucial to pay attention to the onset or change of MR at the follow-up. The absence of MR before treatment is no guarantee for the absence of this complication during the follow-up period. In our study, none of the surgical or transcatheter methods were superior to each other in the development of new MR as a complication of the treatment.

Also, regarding AR change there is no consensus over the choice of surgery or percutaneous method for pmVSD closure when the VSD is associated with mild to moderate AR. Although the emphasis is on the surgical method in these patients, the transcatheter method has recently been introduced [28]. An aortic rim less than 2 mm, aortic leaflet prolapse, and more than mild AR make the transcatheter repair a challenging therapy. The mechanism of new AR after surgical closure of pmVSDs is due to the anatomical abnormality. The lack of muscle support under the aortic valve can lead to leaflet herniation, and the "venturi effect" of the blood circulation during systole might pull the cusps toward the defect [29]. The mechanism of AR induction following the transcatheter method is the impingement of the device on the aortic cusps or interference of the device with the movement of the leaflets [27]. If the aortic rim is less than 2 mm in non-aneurysmal VSDs, the left ventricular disk of the Amplatzer duct occluders can interfere with the aortic valve [2].

Shijun HU and colleagues conducted a study using the transcatheter method to close pmVSDs with an aortic rim less than 2 mm in the pediatric age group. The enrolled patients were with or without AR and with or without aortic valve prolapse. They showed that VSD device occlusion with eccentric zero-edge pmVSD Amplatzer is possible and safe. AR decreased or did not alter during 3 to 36 months of follow-up [3].

According to another study by Guan-Liang Chen, 65 patients aged 3–14 years with mild AR and aortic valve prolapse were evaluated. The results of transcatheter VSD closure in these patients using eccentric zero-edge pmVSD Amplatzer were acceptable. During one-year follow-up, AR decreased in 62% of patients and aortic prolapse reduced in 34%, and only two patients had an increase in the degree of AR. Due to the few complications reported in this study, the author recommended the transcatheter method to treat these patients [28].

In another study, 103 children with subaortic VSD, including 27% pretreatment AR underwent surgery. Less than 5% had some degrees of AR at the follow-up, and AR increased only in 1 patient [29].

In our study, four patients had pm to subaortic VSDs, closed by the transcatheter method. One of them had no AR before and after the treatment. The other three patients had trivial to mild AR before the treatment, which did not change during the follow-up.

According to previous articles [28] and the present study, the improvement of AR is not entirely predictable in both methods. However, we might conclude that because the AR degree in most patients decreased or unchanged, the transcatheter method can be used if the AR degree and prolapse are mild to moderate.

It is important to mention that, we used different VSD Amplatzers: pm, eccentric and muscular types of VSD Amplatzers and Occlutech brand Amplatzer duct occluders, while, some researchers like Zhao et al. and Haddad et al. used Amplatzer duct occluder type II for pmVSD closure. This device is softer, more flexible, and has a lower profile, facilitating its deployment. Further, the adaptation of this device with the shape of VSDs and no interference with aortic valve cusps is very beneficial [30]. Some used Nit-occlud Le VSD coil with the main advantage of lower AR, but it had the foremost disadvantage of residual shunt and hemolysis [31].

Also, it is worth noting that we evaluated the antegrade method for Amplatzer insertion, while the retrograde method has recently been used, especially for insertion of Amplatzer duct occluder type II. The retrograde approach reduces the possibility of tricuspid valve injuries, but it has no effect on the number and extent of aortic valve damage [2, 30].

## Conclusion

This study was designed to compare the development and improvement of MR and AR in surgical and transcatheter VSD closure methods. In cases with pmVSD and mild to moderate MR without mitral valve anomaly, the presence of regurgitation does not necessitate surgical mitral valve repair. The presence of VSD and trivial to mild AR or mild aortic leaflet prolapse are not definite indications for aortic valve repair. It seems that the success rate and the validity of percutaneous VSD closure in terms of MR and AR changes are equal to surgical repair. VSD closure with both methods in cases without regurgitation may almost equally lead to the onset of MR or AR.

### Limitations

In our study, the size of VSDs in the surgical and transcatheter groups was not the same, and the larger and multicenter prospective studies should be conducted in which the size of VSDs and the age and weight of the cases are matched.

## Supplementary Information

**Additional file 1**. The percutaneous antegrade technique of the pmVSD closure.

## Data Availability

also, concerning data availability, we state that the data used and analyzed during the current study are available from the corresponding author on reasonable request. Data sharing applies to this article, and datasets were generated and analyzed during the current study, and data sharing is allowed.

## Abbreviations

AR: Aortic regurgitation
MR: Mitral regurgitation
PAH: Pulmonary arterial hypertension
pmVSD: Perimembranous ventricular septal defects
Qp: Pulmonary flow
Qs: Systemic flow
SD: Standard deviation
TR: Tricuspid regurgitation
VSD: Ventricular septal defects
