# Correction: Mitral and aortic valve regurgitation following surgical and transcatheter perimembranous ventricular septal defect closure in children and adolescents: midterm outcomes

**DOI:** 10.1186/s12872-022-02820-5

**Published:** 2022-08-23

**Authors:** Mohammadreza Edraki, Mohammadjavad Nobakhti, Amir Naghshzan, Hamid Amoozgar, Ahmadali Amirghofran, Bahram Ghasemzadeh, Elahe Nirooie, Nima Mehdizadegan, Hamid Mohammadi, Kambiz Keshavarz

**Affiliations:** 1 Cardiovascular and Neonatology Research Center, School of Medicine, Shiraz University of Medical Sciences, Shiraz, Iran; 2 Cardiac Surgery Department, School of Medicine, Shiraz University of Medical Sciences, Shiraz, Iran; 3 Social Determinants of Health Research Center, Yasuj University of Medical Sciences, Yasuj, Iran; 4 Namazi Hospital, Shiraz, Iran

## Correction to: BMC Cardiovascular Disorders (2022) 22:315 10.1186/s12872-022-02757-9

Following the publication of the original article [1], the author name ‘Mohammadjavad Nobakhti’ has been misspelled as ‘Mohammadjavad Nobahkti’.

The original article has been corrected.
